# Multicenter phase II clinical trial of nilotinib for patients with imatinib-resistant or -intolerant chronic myeloid leukemia from the East Japan CML study group evaluation of molecular response and the efficacy and safety of nilotinib

**DOI:** 10.1186/2050-7771-2-6

**Published:** 2014-03-20

**Authors:** Naoto Takahashi, Masatomo Miura, Jun Kuroki, Kinuko Mitani, Atsushi Kitabayashi, Osamu Sasaki, Hideo Kimura, Kiyotoshi Imai, Norifumi Tsukamoto, Hideyoshi Noji, Takeshi Kondo, Mutsuhito Motegi, Yuichi Kato, Masayuki Mita, Hajime Saito, Chikashi Yoshida, Yoshihiro Torimoto, Tomofumi Kimura, Yuji Wano, Jun Nomura, Satoshi Yamamoto, Ko Mayama, Riko Honma, Tomohiro Sugawara, Shinji Sato, Atsushi Shinagawa, Maiko Abumiya, Takenori Niioka, Hideo Harigae, Kenichi Sawada

**Affiliations:** 1 Department of Hematology, Nephrology, and Rheumatology, Akita University Graduate School of Medicine, 1-1-1 Hondo, 010-8543 Akita, Japan; 2 Department of Pharmacology, Akita University Hospital, Akita, Japan; 3 Department of Internal Medicine, Yuri Kumiai General Hospital, Yurihonjo, Japan; 4 Department of Hematology and Oncology, Dokkyo Medical University School of Medicine, Mibu-machi, Japan; 5 Department of Internal Medicine, Akita Kumiai General Hospital, Akita, Japan; 6 Division of Hematology, Department of Internal Medicine, Miyagi Cancer Center, Natori, Japan; 7 Department of Hematology, Kita-Fukushima Medical Center, Date, Japan; 8 Department of Hematology, Sapporo Hokuyu Hospital, Sapporo, Japan; 9 Oncology Center, Gunma University Hospital, Maebashi, Japan; 10 Department of Cardiology and Hematology, Fukushima Medical University, Fukushima, Japan; 11 Stem Cell Transplantation Center, Hokkaido University Hospital, Sapporo, Japan; 12 Department of Internal Medicine, Senboku Kumiai General Hospital, Daisen, Japan; 13 Department of Neurology, Hematology, Metabolism, Endocrinology and Diabetology, Yamagata University Faculty of Medicine, Yamagata, Japan; 14 Department of Cardiology and Hematology, Shirakawa Kosei General Hospital, Shirakawa, Japan; 15 Department of Internal Medicine, Mito Chuo Hospital, Mito, Japan; 16 Department of Hematology, Mito Medical Center, Mito, Japan; 17 Oncology Center, Asahikawa Medical College Hospital, Asahikawa, Japan; 18 Division of Hematology, Department of Internal Medicine, Suifu Hospital, Mito, Japan; 19 Department of Hematology, Iwate Prefectural Central Hospital, Morioka, Japan; 20 Department of Internal Medicine, NTT East Japan Tohoku Hospital, Sendai, Japan; 21 Department of Hematology, Sapporo City General Hospital, Sapporo, Japan; 22 Department of Gastroenterology and Hematology, Hirosaki University Graduate School of Medicine, Hirosaki, Japan; 23 Department of Hematology, Yamagata Prefectural Central Hospital, Yamagata, Japan; 24 Department of Internal Medicine, Osaki Citizen Hospital, Osaki, Japan; 25 Division of Hematology, Department of Internal Medicine, Okitama Public General Hospital, Higashi Okitama, Japan; 26 Division of Hematology, Department of Internal Medicine, Hitachi General Hospital, Hitachi, Japan; 27 Department of Hematology and Rheumatology, Tohoku University Graduate School of Medicine, Sendai, Japan

**Keywords:** Chronic myeloid leukemia, Nilotinib, *BCR-ABL1* mutation, Major molecular response, Hyperbilirubinemia, Uridine diphosphate glucuronosyltransferase

## Abstract

**Background:**

Nilotinib is a second-generation tyrosine kinase inhibitor that exhibits significant efficacy as first- or second-line treatment in patients with chronic myeloid leukemia (CML). We conducted a multicenter Phase II Clinical Trial to evaluate the safety and efficacy of nilotinib among Japanese patients with imatinib-resistant or -intolerant CML-chronic phase (CP) or accelerated phase (AP).

**Results:**

We analyzed 49 patients (33 imatinib-resistant and 16 imatinib-intolerant) treated with nilotinib 400 mg twice daily. The major molecular response (MMR) rate was 47.8% at 12 months among 35 patients who did not demonstrate an MMR at study entry. Somatic *BCR-ABL1* mutations (Y253H, I418V, and exon 8/9 35-bp insertion [35INS]) were detected in 3 patients at 12 months or upon discontinuation of nilotinib. Although 75.5% of patients were still being treated at 12 months, nilotinib treatment was discontinued because of progressing disease in 1 patient, insufficient effect in 2, and adverse events in 9. There was no statistically significant correlation between MMR and trough concentrations of nilotinib. Similarly, no correlation was observed between trough concentrations and adverse events, except for pruritus and hypokalemia. Hyperbilirubinemia was frequently observed (all grades, 51.0%; grades 2–4, 29%; grades 3–4, 4.1%). Hyperbilirubinemia higher than grade 2 was significantly associated with the uridine diphosphate glucuronosyltransferase (*UGT*)*1A9* I399C/C genotype (*P* = 0.0086; Odds Ratio, 21.2; 95% Confidence Interval 2.2–208.0).

**Conclusions:**

Nilotinib was efficacious and well tolerated by patients with imatinib-resistant or -intolerant CML-CP/AP. Hyperbilirubinemia may be predicted before nilotinib treatment, and may be controlled by reducing the daily dose of nilotinib in patients with *UGT1A9* polymorphisms.

**Trial registration:**

clinicaltrials.gov: UMIN000002201

## Background

Imatinib is used as a first-line therapy for newly diagnosed Philadelphia chromosome-positive chronic myeloid leukemia (Ph + CML) [1,2]; however, some patients fail to respond or become intolerant to this treatment [3]. Nilotinib is a second-generation tyrosine kinase inhibitor (2G-TKI) with higher selectivity and more potent inhibitory effects on the breakpoint cluster region-Abelson 1 (BCR-ABL1) tyrosine kinase than imatinib [4,5]. Several studies have shown hematologic and cytogenetic responses to nilotinib in patients with imatinib-resistant or -intolerant CML [6-11]. Although point mutations in *BCR-ABL1* are a major cause of imatinib-resistance, nilotinib is effective in patients with known point mutations in this oncogene, with the exception of the T315I mutation [3]. However, the frequency or profile of *BCR-ABL1* point mutations has not been determined in daily practice when treating Japanese patients with imatinib-resistant CML in chronic phase (CP) or accelerated phase (AP).

Hyperbilirubinemia is one of the adverse events (AEs) caused by nilotinib. Recently, Giles et al. reported a correlation between hyperbilirubinemia and nilotinib trough concentrations in patients treated with nilotinib [12]. Moreover, nilotinib is not a substrate for uridine diphosphate glucuronosyltransferase 1A1 (UGT1A1) enzymes, but an inhibitor of human UGT1A1 *in vitro*[13], and CML patients with the UGT1A1*28 polymorphism show an increased risk of nilotinib-induced hyperbilirubinemia [14]. In Japanese cancer patients, UGT1A1*28, UGT1A9*1b, UGT1A1*6 (211G > A), and UGT1A1*60 (3279 T > G) are closely associated with the *UGT1A9* IVS1 + 399 (I399C > T) polymorphism, and linkage of I399C > T with these variants has been shown to affect irinotecan metabolism [15]. However, we are not aware of any studies that have examined a correlation between hyperbilirubinemia and the *UGT1A9* I399C > T polymorphism.

The goals of the present study were as follows: 1) to determine the major molecular response rate (MMR) at 12 months of twice daily (BID) treatment with 400 mg nilotinib in patients with imatinib-resistant/intolerant CML-CP or -AP; 2) to evaluate molecular responses associated with *BCR-ABL1* mutation status or plasma concentrations of nilotinib; and 3) to evaluate the safety of administering 400 mg nilotinib BID, including hyperbilirubinemia development, based on plasma concentrations of nilotinib or polymorphisms of *UGT1A1* and *UGT1A9*.

## Results

### Patients and treatment

Between March 13, 2009 and January 12, 2011, 51 Japanese patients were recruited, and 49 patients (CML-CP, n = 45; CML-AP, n = 4) were included in the study. Two patients were excluded because they withdrew informed consent. The cut-off date for overall survival (OS) was January 11, 2013 (24 months after the last patient enrolled).

The demographic and baseline disease characteristics of the patients are described in Table 1. The median age was 62 years. The ratio of men to women was 27:22. Thirty-three (67.3%) and 16 (32.7%) patients were imatinib-resistant or imatinib-intolerant, respectively. The median duration of CML in patients was 46.0 months. The median duration of prior imatinib treatment was 43.4 months. Patients were treated with interferon (9 patients; 18.4%) with or without hydroxyurea (8 patients; 16.3%) prior to imatinib therapy. Twenty-three patients (46.9%) showed a complete cytogenetic response (CCyR), and 14 patients (28.6%) showed MMR at the time of entry into the study. Five patients (15.2%) had an additional chromosomal abnormality, and 6 patients (12.2%) had *BCR-ABL1* kinase domain mutations at baseline.

**Table 1:** Baseline patient characteristics

**Parameters**	**Value**
Median age, y (range)	62 (19–84)
Men/women	27/22
Chronic phase/accelerated phase	45/4
Median duration of CML, months (range)	46.0 (3.6-208.2)
Median duration of imatinib therapy, months (range)	43.4 (3.1-101.9)
Imatinib-resistant/-intolerant	33/16
Prior TKI therapy	
Imatinib/dasatinib/bosutinib	49/4/2
Other prior therapy	
Interferon/hydroxyurea/AraC/BU	9/8/1/1
Cytogenetic response at baseline, Ph +%	
>95/>65-95/>35-65/>0-36/0	14/6/4/2/23
*BCR-ABL1* level at baseline, IS%	
>10/>1-10/>0.1-1/**≤**0.1	18/7/10/14
Additional chromosomal abnormality, n (%)	5 (15.2)
*BCR-ABL1* mutations, n (%)	6 (12.2)

CML, chronic myeloid leukemia; mo, months; TKI, tyrosine kinase inhibitor; AraC, cytarabine; BU, busulfan; Ph +%, % of Philadelphia chromosome-positive patients; IS%, international scale %.

### Molecular response

**Figure 1:**
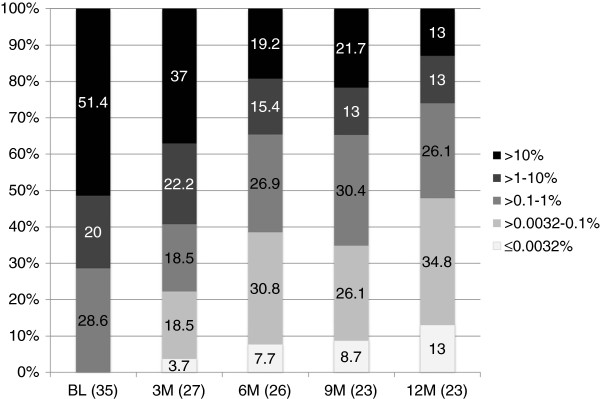
**Molecular responses in patients with imatinib-resistant and imatinib-intolerant CML treated with nilotinib.** The molecular response was evaluated according to the international scale of standardized quantitative real-time polymerase chain reaction at the beginning of the study and every 3 months thereafter. The X-axis shows the time-point and patient number for molecular response evaluation. The Y-axis shows the percentage of patients who achieved a molecular response.

Of the 49 patients on trial, 35 did not demonstrate an MMR at study entry and were evaluable for response, using the international scale of standardized quantitative real-time polymerase chain reaction (IS-PCR). The rates of MMR in the evaluable patients were 38.5% and 47.8% at 6 and 12 months, respectively, and the rates of MR4.5 were 7.7% and 13.0% at 6 and 12 months, respectively (Figure 1). The rates of *BCR-ABL1* transcript levels (BCR-ABL^IS^) of <1% and <10% at 12 months were 74% and 87%, respectively.

**Figure 2:**
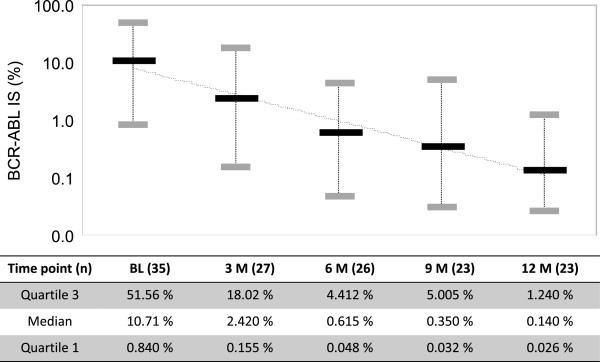
**Kinetics of *BCR-ABL1 *expression.** The black bars indicate the median. The gray bars indicate quartiles 1 and 3. The X-axis shows the time-point and patient number for evaluation of molecular responses. The Y-axis shows *BCR-ABL1* transcript levels per the international scale of standardized quantitative real-time polymerase chain reaction.

The cumulative MMR rates at 12 months were 62.5%, 33.3%, and 24.8% in patients with baselines >0.1–1%, >1–10%, and >10%, respectively. These data represented a significant difference between patients with baseline >0.1–1% and those with baseline >10% (log-rank test, *P* = 0.0372). The median *BCR-ABL1* transcript levels (BCR-ABL^IS^) reduced significantly from 10.71% to 0.14% by 12 months in a time dependent manner (Figure 2).

### *BCR-ABL1* mutations and nilotinib trough concentrations

Using the direct sequencing identification method (DS), 5 *BCR-ABL1* mutations (M244V, F317L, N358D, F359V, and E459K) were detected in 6 patients (12.2%) at baseline. However, M244V, N358D, and E459K were undetectable after nilotinib treatment at 12 months. In contrast, new somatic *BCR-ABL1* mutations (Y253H, I418V, and exon 8/9 35-bp insertion [35INS]) were detected in 3 patients (6.1%) at 12 months. These 3 patients did not achieve MMR. The somatic *BCR-ABL1* mutation (T315I) was not detected.

Nilotinib trough plasma concentrations were measured by high-performance liquid chromatography (HPLC) methods using blood collected at 3 months. Among 20 patients who continued treatment with nilotinib for 12 months and did not show somatic *BCR-ABL1* mutations, trough concentrations (median, 25% to 75%) were increased in patients who achieved MMR at 12 months (774.4 ng/mL, 584.0 to 1364.0) compared to patients without MMR (489.7 ng/mL, 317.6 to 922.5). However, the correlation between MMR at 12 months and trough concentrations of nilotinib was not statistically significant (*P* = 0.261).

### Patient status

**Figure 3:**
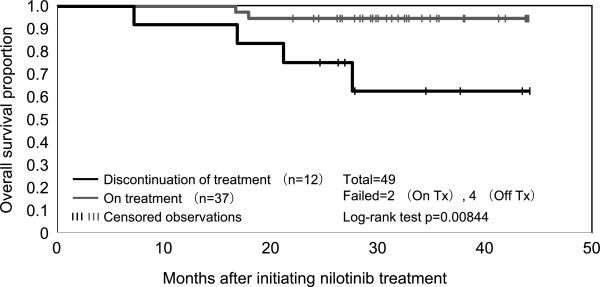
**Overall survival of patients on present or discontinued treatment after 12 months.** OS was measured from the date of initial nilotinib administration to the date of death, with a minimum follow-up of 24 months. OS was estimated using the Kaplan-Meier method and compared between groups using the stratified log-rank test. There was a significant difference between the 2 groups (log-rank test, *P* = 0.00844).

The disposition of patients at 12 months is summarized in Table 2. Thirty-seven patients (75.5%) were still being treated with nilotinib treatment, while treatment was discontinued for 12 patients (24.5%). AEs were the most frequent reasons for terminating treatment (9 patients; 18.4%). CML-CP progressed in 1 patient with an F317L mutation at baseline. The median actual dose intensity (total nilotinib dose/treatment time) was 651 mg/day. The estimated 36-month OS was 95% in the 37 patients on treatment and 60% in the 12 patients for which treatment was terminated. These data represented a significant difference between the two groups (Figure 3; log-rank test, *P* = 0.00844).

**Table 2:** Disposition of patients at 12 months

**Status**	**N = 49**
On treatment	37 (75.5%)
Treatment discontinued	12 (24.5%)
Adverse events	9 (18.4%)
Thrombocytopenia	5 (10.2%)
Hyperbilirubinemia	2 (4.1%)
Heart disease	1 (2.0%)
Headache	1 (2.0%)
No molecular response	2 (4.1%)
Disease progression	1 (2.0%)
Median actual dose intensity, mg/day (range)	651 (198–800)

### Safety

Nilotinib therapy was well tolerated. Although severe nonhematologic AEs were infrequent, 1 patient presenting with grade 3/4 headache and 1 with ischemic heart disease discontinued nilotinib therapy. Grade 3/4 hematologic AEs included anemia (14.3%), neutropenia (28.6%), and thrombocytopenia (18.4%). Although hematologic AEs were managed with dose interruptions or reductions, 5 patients discontinued nilotinib because of repeated or prolonged grade 3/4 thrombocytopenia. Although corrected QT interval (QTc) prolongation at all grades was found in only three patients (6.1%), grade 3/4 QTc prolongation was not detected. QTc prolongation was also easily managed with dose interruptions or reductions, and no patients discontinued treatment because of this AE. Moreover, no episodes of torsades de pointes or death due to arrhythmias were noted. However, 1 patient discontinued nilotinib therapy because of ischemic heart disease without QTc prolongation.

Grade 3/4 elevations of total bilirubin occurred in 2 patients (4.1%), and their nilotinib therapy was discontinued because of repeated hyperbilirubinemia despite dose interruptions or reductions. Although other biochemical abnormalities were generally mild, transient, and easily managed with dose interruptions or reductions, hyperbilirubinemia was frequently observed (all grades, 51.0%; grades 2–4, 29%). There was no statistically significant correlation between AEs and trough concentrations of nilotinib except for pruritus (*P* = 0.0440) and hypokalemia (*P* = 0.0221).

The *UGT1A1* and *UGT1A9* polymorphisms were analyzed in all patients to identify a correlation with laboratory abnormalities related to liver function. Although we did not observe a significant correlation between hyperbilirubinemia and *UGT1A1 genotype* (poor metabolizers with UGT1A1*6/*28, *27/*28, or *28/*28 vs. extensive metabolizers with *UGT1A1* *1/*1, *1/*6, *1/*27, or *1/*28)*,* there was a significant correlation with the *UGT1A9* I399C/C genotype (*P* = 0.028) (Table 3). This genotype was observed in 33% of patients with hyperbilirubinemia higher than grade 2. The *UGT1A9* I399C/C genotype was a statistically independent factor according to the results of stepwise forward selection multiple regression analysis (*P* = 0.0086, Odds Ratio, 21.2; 95% Confidence Interval, 2.2–208.0).

**Table 3:** **Liver dysfunction and *UGT1A1/1A9 *** **polymorphisms**

**Liver test**	**Grades 2-4**	***UGT1A1***			***UGT1A9*** **I399C > T**		
		**Poor metabolizers**	**Extensive metabolizers**	***P*-value**	**C/T + T/T**	**C/C**	***P*-value**
ALT	No	3	41	0.359	39	4	0.111
	Yes	1	4		3	2	
AST	No	3	44	0.158	41	5	0.237
	Yes	1	1		1	1	
T.Bil	No	1	35	0.052	34	2	0.028
	Yes	3	10		8	4	
Nilotinib C_0_	Median	687.6	1042.0	0.577	977.5	644.0	0.685
	Range (25–75)	(336.1-1906.6)	(614.9-1472.8)		(544.0-1473.0)	(623.0-2292.0)	

Poor metabolizers, *UGT1A1* *6/*28, *27/*28, or *28/*28; Extensive metabolizers, *UGT1A1* *1/*1, *1/*6, *1/*27, or *1/*28; C_0_, trough concentration.

## Discussion

The present study revealed that nilotinib therapy was effective in Japanese patients with CML-CP/AP who have developed imatinib resistance or intolerance. Approximately 50% of the patients in this Phase II trial achieved MMR, and the estimated OS at 36 months for patients on treatment was 95%. Responses were more likely in patients with BCR-ABL^IS^ ≤1% at baseline than in those with BCR-ABL^IS^ >1%. In a phase II trial of nilotinib for imatinib resistance or intolerance reported by Kantarjian et al. [9], MMR was achieved in 32% (43 of 134) of patients at 12 months. The lower MMR reported in that study as compared to the rate observed in the present study might be explained by differences in response at baseline. The CCyR at baseline observed by Kantarjian et al. [9] was 3%; in comparison, 26% (9 of 35 patients) showed this response in our trial. In the Expanding Nilotinib Access in Clinical Trials (ENACT), the molecular responses in a subset of French patients (n = 168) every 3 months were evaluated. Thirty-seven percent achieved MMR, and 20% achieved MR4.5 by 12 months [10]. In a phase I/II study of nilotinib treatment of Japanese patients with imatinib-resistant or -intolerant CML or relapsed/refractory Ph + acute lymphoblastic leukemia (ALL), 16 patients with CML-CP were assessed using IS-PCR to detect molecular responses every 3 months [6]. The level of *BCR-ABL* transcription gradually decreased from baseline, with a 1-log reduction at 6 months and a 2-log reduction at 12 months. Our findings are consistent with these observations and suggest that nilotinib is highly active in patients with imatinib-resistant or -intolerant CML-CP/AP.

We detected 5 *BCR-ABL* mutations (M244V, F317L, N358D, F359V, and E459K) in 6 patients at baseline screening. Among these patients, M244V, N358D, and E459K were not detected after nilotinib treatment at 12 months, and 3 patients with these mutations achieved MMR. In contrast, patients with F317L and F359V + E459K mutations showed poor responses to nilotinib treatment, and 1 patient with F317L experienced disease progression. The remaining patient with F359V + E459K mutations did not respond to nilotinib, and treatment was discontinued because of grade 3 thrombocytopenia. A previous study showed that patients with F317L or F359V mutations are resistant to nilotinib [16,17]. The present study also suggests that these mutations at baseline may be associated with less favorable responses to nilotinib.

We detected 3 new mutations in 3 patients after they started nilotinib treatment (Y253H, I418V, and exon 8/9 35-bp insertion). Although the Y253H mutation was less sensitive to nilotinib in an *in vitro* cell viability assay (IC_50_ > 150 nM) [17], the level of *BCR-ABL1* transcripts in the Y253H patient in our trial decreased from 8.06% to 0.30%. Qin et al. [18] reported the I418V mutation in a Chinese patient who progressed to blast phase while on imatinib therapy. Although a patient with the somatic I418V mutation in our trial was in second chronic phase at baseline, the disease did not progress. However, the levels of the *BCR-ABL1* transcript were maintained from 59.36% at baseline to 50.21% at 12 months. Another rare mutation of *BCR-ABL1* is 35INS, which is a 35-bp insertion between ABL kinase domain exons 8 and 9 [19]. This insertion results in a frameshift, leading to the addition of 10 residues and truncation of 653C-terminal residues due to early termination [20]. One patient with 35INS in our trial showed a mild reduction in the level of *BCR-ABL1* transcripts from 10.71% at baseline to 2.89% at 12 months. O’Hare et al. reported that 35INS is kinase-inactive and does not contribute to TKI resistance [21]. Patients who did not achieve MMR and harbored somatic mutations showed relatively poor responses to nilotinib. However, a mutation (e.g. 35INS) might not be causally related, suggesting the possibility of genomic instability, and that other genetic abnormalities might contribute to TKI resistance. Conversely, the T315I mutation, which is known to cause resistance to imatinib and nilotinib, was not detected in the present study.

In the Evaluating Nilotinib Efficacy and Safety in Clinical Trials-newly diagnosed (ENESTnd) patients, the occurrence of all-grade total bilirubin elevation was significantly higher in patients with higher nilotinib exposure [22]. Although we could not identify a correlation between nilotinib trough concentrations and hyperbilirubinemia in the present study, nilotinib trough concentration has been associated with the occurrence of all-grade elevations in total bilirubin [12]. However, to our knowledge this is the first report of a significant correlation between hyperbilirubinemia and the *UGT1A9* I399C/C genotype.

UGT1A1 catalyzes gluconidation of hepatic bilirubin in humans [23]. The presence of 7-TA nucleotide repeats in the (TA)nTAA promoter region of *UGT1A1* (UGT1A1*28) leads to decreased expression of this gene, resulting in high plasma bilirubin levels that form the basis for Gilbert’s syndrome [24]. Because nilotinib is a potent noncompetitive inhibitor of human UGT1A1 activity [13], the UGT1A1*28 polymorphism increases the risk of nilotinib induced-hyperbilirubinemia [14,25]. These observations suggest that nilotinib-associated hyperbilirubinemia is very likely the result of inhibition of UGT1A1 activity, combined with genetic defects in *UGT1A1*. Although, UGT1A9 is also an enzyme of the glucuronidation pathway that transforms small lipophilic molecules, such as steroids, bilirubin, hormones, and drugs, into water-soluble, excretable metabolites, we are not aware of any reports indicating that nilotinib-induced hyperbilirubinemia is related to the *UGT1A9* I399C/C genotype. It has been shown that the enzyme function of the *UGT1A9* I399C > T genotype does not contribute to variability in UGT1A9 activity [26]. On the other hand, Saito et al. reported the role of *UGT1A9* I399C > T in SN-38 glucuronidation in a study of 177 Japanese cancer patients administered irinotecan. Haplotype analysis showed that 98% of *UGT1A9* I399C alleles was linked to low-activity genotypes, either UGT1A1*6*,* *28*, or* *60 [15]. Our study suggests that hyperbilirubinemia is associated with the *UGT1A9* I399C/C genotype. For Japanese patients with CML, hyperbilirubinemia may be predicted before nilotinib treatment and controlled by reducing the daily dose of nilotinib in patients with *UGT1A9* polymorphisms.

## Conclusions

The results of our study suggest that nilotinib is generally well tolerated by patients and effectively treats imatinib-resistant or -intolerant CML-CP/AP.

## Methods

### Eligibility criteria

Twenty-seven institutions in the East Japan CML study group (EJCML) participated. Patients with Ph + CML-CP/AP who were at least 18 years of age were eligible if they were imatinib-resistant or -intolerant, demonstrated adequate performance status (World Health Organization [WHO] Performance Score [PS] <2), and had normal hepatic, renal, and cardiac functions. Patients were excluded if they exhibited any of the following characteristics: blast phase, QTc >450 ms, and the T315I mutation. Imatinib resistance was defined as an incomplete hematologic response at or after 3 months, no MCyR at 6 months, no CCyR at 12 months, or no MMR at 18 months, according to the criteria of failure and suboptimal response of European LeukemiaNet [3]. Imatinib intolerance was defined as a lack of optimal response because of a grade 3/4 imatinib-related AE, or because of a persistent grade 2 imatinib-related AE, despite optimal supportive care, that persisted for more than 1 month or recurred more than 3 times following reduction of the dose of imatinib.

### Clinical trial design and objectives

The study was conducted in accordance with the principles of the Declaration of Helsinki. Informed consent was written by all patients according to institutional guidelines. The study was approved by all institutional review boards and registered with ClinicalTrials.gov (number UMIN000002201). The primary objective of this phase II, single-treatment arm, open-label study was to determine the incidence of MMR at 12 months in patients with imatinib-resistant/intolerant CML-CP/AP who were treated with nilotinib. The molecular response was evaluated according to IS-PCR upon study entry and every 3 months thereafter. Secondary objectives were to evaluate the relationship between the molecular response and *BCR-ABL1* point mutations or plasma concentrations of nilotinib, and between the safety profile of nilotinib and plasma concentrations of the drug and or *UGT 1A1/1A9* polymorphisms.

### Dosing regimen and concomitant therapy

Nilotinib was administered orally at 400 mg BID, at approximately 12-h intervals. Food was not consumed with the drug, or for at least 2 h before and 1 h after the drug was ingested. The concomitant administration of a strong inhibitor, including grapefruit juice, or inducer of CYP3A, was not allowed in the study.

### Molecular response

IS-PCR was performed in a central laboratory (BML Inc., Kawagoe, Japan) using the MolecularMD One-Step qRT-PCR BCR-ABL kit (MolecularMD, Cambridge, MA). An MMR was defined as a 3-log reduction in the level of the *BCR-ABL1* transcript using IS-PCR (BCR-ABL^IS^ 0.0032–0.1%). At least 10,000 control genes (*ABL1*) were required for a sample to be classified as adequate. MR4.5 was defined as ≥4.5-log reduction in the levels of the *BCR-ABL1* transcript (BCR-ABL^IS^ ≤0.0032%) in peripheral blood samples, and at least 32,000 control genes were required for a sample to be classified as adequate. Patients with MMR at baseline were considered not eligible and were excluded from the analysis of response rates.

### Measurement of nilotinib concentration and Genotyping

Nilotinib trough concentrations (C_0_) were determined using HPLC as described previously [27]. *BCR-ABL1* mutations were analyzed by the DS method as previously described [28]. Genotyping of UGT1A1*6, *27 and *28 was performed using PCR-restriction fragment length polymorphism as described [29,30]. The analysis results obtained from PCR-RFLP were confirmed using a fully automated single nucleotide polymorphism (SNP) detection system (prototype i-densy™, ARKRAY Inc., Kyoto, Japan). The *UGT1A9* I399C > T (relative to the end of *UGT1A9* exon 1) polymorphism was genotyped by direct sequencing using a PCR procedure as previously described [31]. All genotype frequencies were tested for their consistency with Hardy-Weinberg equilibrium.

### Safety analyses

Safety was assessed by determining the frequency and severity of AEs, which included hematologic and biochemical laboratory tests, vital signs, physical examinations (including body weight), WHO PS cardiac function tests (12-lead electrocardiogram, cardiac enzyme test, and echocardiography), and chest X-rays. AEs were graded according to the National Cancer Institute Common Terminology Criteria for AEs (version 3.0).

### Statistical analyses

Statistical analyses were performed using SPSS statistical software (version 17.0, SPSS Japan Inc., Tokyo, Japan). Data were presented as number or median values (range or 25% to 75%). Differences in the various parameters between groups were evaluated using the Mann–Whitney U or chi-square tests. Time to MMR was measured from the date of initial nilotinib administration to the date of MMR during the study. OS was measured from the date of initial nilotinib administration to the date of death with a minimum follow-up of 24 months. OS and the cumulative incidence of MMR were estimated using the Kaplan-Meier method and compared between groups using the stratified log-rank test. Stepwise forward selection multiple regression analysis was performed to determine the effect of the variables examined in univariate analysis. A *P* value of <0.05 was considered significant.

## Abbreviations

CML: Chronic myeloid leukemia; Ph: Philadelphia chromosome; CP: Chronic phase; AP: Accelerated phase; UGT: Uridine diphosphate glucuronosyltransferase; MMR: Major molecular response; 2G-TKI: Second-generation tyrosine kinase inhibitor; BCR-ABL1: Breakpoint cluster region-Abelson 1; AEs: Adverse events; OS: Overall survival; CCyR: Complete cytogenetic response; MCyR: Major cytogenetic response; IS-PCR: The international scale of standardized quantitative real-time polymerase chain reaction; DS: The direct sequencing identification method; 35INS: Exon8/9 35-bp insertion; QTc: Corrected QT interval; HPLC: High-performance liquid chromatography; WHO: World Health Organization; PS: Performance score.

## Competing interests

NT, KM, TK, YK, CY, SS, HH and KS receive research funding and honoraria for lectures from Novartis Pharmaceuticals, and Bristol-Myers Squibb. The other authors declare that they have no competing interests.

## Authors’ contributions

NT and KS designed and performed the research. NT wrote the paper. TN, MA, and MM performed the pharmacokinetic and pharmacogenetic analyses. NT, TN, and MM analyzed the data and prepared the figures. JK, KM, AK, OS, HK, KI, NT, HN, TK, MM, YK, KT, MM, HS, CY, YT, TK, YW, JN, SY, KM, RH, TS, SS, AS, and HH participated in the study design and data analysis. All authors read and approved the final manuscript.
